# Tyrosine phosphatase SHP2 in ovarian granulosa cells balances follicular development by inhibiting PI3K/AKT signaling

**DOI:** 10.1093/jmcb/mjac048

**Published:** 2022-08-24

**Authors:** Xiaoli Wei, Lanping Zheng, Yingpu Tian, Haibin Wang, Youqiang Su, Gensheng Feng, Chao Wang, Zhongxian Lu

**Affiliations:** School of Pharmaceutical Sciences, State Key Laboratory of Cellular Stress Biology, Xiamen University, Xiamen 361005, China; School of Pharmaceutical Sciences, State Key Laboratory of Cellular Stress Biology, Xiamen University, Xiamen 361005, China; School of Pharmaceutical Sciences, State Key Laboratory of Cellular Stress Biology, Xiamen University, Xiamen 361005, China; Fujian Provincial Key Laboratory of Reproductive Health Research, Medical College of Xiamen University, Xiamen 361102, China; State Key Laboratory of Reproductive Medicine, Nanjing Medical University, Nanjing 211166, China; Department of Pathology, Division of Biological Sciences, University of California at San Diego, La Jolla, CA 92093, USA; State Key Laboratory of Agrobiotechnology, College of Biological Sciences, China Agricultural University, Beijing 100193, China; School of Pharmaceutical Sciences, State Key Laboratory of Cellular Stress Biology, Xiamen University, Xiamen 361005, China; Fujian Provincial Key Laboratory of Reproductive Health Research, Medical College of Xiamen University, Xiamen 361102, China

**Keywords:** tyrosine phosphatase, SHP2, granulosa cell, follicular development, PI3K/AKT signaling

## Abstract

In mammals, the growth and maturation of oocytes within growing follicles largely depends on ovarian granulosa cells (GCs) in response to gonadotropin stimulation. Many signals have been shown to regulate GC proliferation and apoptosis. However, whether the tyrosine phosphatase SHP2 is involved remains unclear. In this study, we identified the crucial roles of SHP2 in modulating GC proliferation and apoptosis. The production of both mature oocytes and pups was increased in mice with *Shp2* specifically deleted in ovarian GCs via *Fshr*-*Cre. Shp2* deletion simultaneously promoted GC proliferation and inhibited GC apoptosis. Furthermore, *Shp2* deficiency promoted, while *Shp2* overexpression inhibited, the proliferation of cultured primary mouse ovarian GCs and the human ovarian granulosa-like tumor cell line KGN *in vitro. Shp2* deficiency promoted follicule-stimulating hormone (FSH)-activated phosphorylation of AKT *in vivo. SHP2* deficiency reversed the inhibitory effect of hydrogen peroxide (H_2_O_2_) on AKT activation in KGN cells. H_2_O_2_ treatment promoted the interaction between SHP2 and the p85 subunit of PI3K in KGN cells. Therefore, SHP2 in GCs may act as a negative modulator to balance follicular development by suppressing PI3K/AKT signaling. The novel function of SHP2 in modulating proliferation and apoptosis of GCs provides a potential therapeutic target for the clinical treatment of follicle developmental dysfunction.

## Introduction

In mammals, continuous folliculogenesis is governed by not only oocytes but also ovarian granulosa cells (GCs). GCs are the major target of gonadotropins, and their proliferation and apoptosis determine the fate of the follicle (Matsuda et al., 2012; Regan et al., 2018). Follicle-stimulating hormone (FSH) initiates and stimulates the proliferation of GCs to develop preovulatory follicles, and then, the luteinizing hormone surge activates ovulation by regulating the terminal differentiation of GCs (Casarini and Crepieux, 2019; Arroyo et al., 2020). In addition, apoptosis of GCs is considered an indication and inducer of follicular atresia (Inoue et al., 2011; Matsuda et al., 2012; Regan et al., 2018). The development and atresia of GCs are governed by FSH, luteinizing hormone, and many paracrine factors, including activin, inhibin, and insulin-like growth factor 1 (IGF1) (Matsuda et al., 2012; Chu et al., 2018; Richani and Gilchrist, 2018; Ipsa et al., 2019; Arroyo et al., 2020). These factors form an extraordinary signal network in GCs and maintain the balance between follicular growth and atresia (Visser et al., 2007; Matsuda et al., 2012).

During folliculogenesis, FSH specifically activates the PI3K/AKT pathway and its target proteins, resulting in follicular growth and maturation (Hunzicker-Dunn et al., 2012; Li et al., 2013). FSH stimulates the transcription and translation of *Cnot6* and *Cnot6l* in GCs, which could function as key effectors of FSH in GCs and trigger the clearance of specific transcripts in GCs during the preantral-to-antral follicle transition (Dai et al., 2021). Hydrogen peroxide (H_2_O_2_) effectively offsets FSH-evoked GC growth by inhibiting PI3K/AKT activation, which involves the initiation of GC death and follicular atresia (Hanukoglu, 2006). Several protein tyrosine phosphatases (PTPs), including phosphatase and tensin homolog (PTEN) and PTP1, were demonstrated to play key roles in follicular development (Reddy et al., 2008; McLaughlin et al., 2014; Idrees et al., 2019; Maidarti et al., 2020). Deletion of *Pten* in oocytes induced abnormal activation of many primordial follicles, resulting in premature ovarian failure (Shimizu et al., 2009). *Pten* deficiency in GCs also enhanced PI3K/AKT activation and significantly elevated GC proliferation and reduced apoptotic susceptibility of GCs (Fan et al., 2008). CRL4–DCAF13 could support oocyte meiotic resumption by targeting the polyubiquitination and degradation of PTEN (Zhang et al., 2020). Thus, protein tyrosine kinases (PTKs) and PTPs are important parts of the cellular signal network in follicular development (McGinnis et al., 2011; Idrees et al., 2020).

Src homology 2 domain-containing protein tyrosine phosphatase 2 (SHP2), encoded by *PTPN11*, is a ubiquitously expressed nonreceptor PTP (Huang et al., 2014). Activated SHP2 can antagonize a variety of signals, including hormones and growth factors, by dephosphorylating active receptors to mediate cell proliferation, apoptosis, and differentiation (Neel et al., 2003). Dysregulation of SHP2 signaling (abnormal protein and gene mutation) is involved in Noonan syndrome, Leopard syndrome, metabolic disorder, and several types of cancer, including breast cancer, liver cancer, and leukemia (Zhang et al., 2015; Wang et al., 2021). In the reproductive system, *Shp2* deficiency in Sertoli cells or spermatogonia disturbs spermatogonial differentiation and spermatocyte meiosis (Puri et al., 2014; Hu et al., 2015; Li et al., 2020). Uterine-specific deletion of *Shp2* was shown to block normal embryo implantation in mice (Ran et al., 2017). Kaur et al. (2012) found that *PTPN11*/*SHP2* was extremely highly expressed in GCs in patients with polycystic ovarian syndrome, who exhibited hyperandrogenism and aberrant folliculogenesis, suggesting that SHP2 in GCs may be involved in the regulation of follicular development. However, there is still a lack of detailed information on how SHP2 works in GCs.

In the present study, we specifically deleted *Shp2* in GCs of developing follicles by *Fshr-Cre* in mice and found that SHP2 deletion increased cell proliferation and inhibited apoptosis of GCs by regulating PI3K/AKT transcriptional activity, thereby promoting oocyte production and improving female fertility. These results suggest that SHP2, as a nonreceptor phosphatase, may balance PTK signals in folliculogenesis and provide an effective therapeutic target for ovarian functional disorders.

## Results

### Conditional knockout of Shp2 in GCs enhances the female fertility of mice

**Figure 1:**
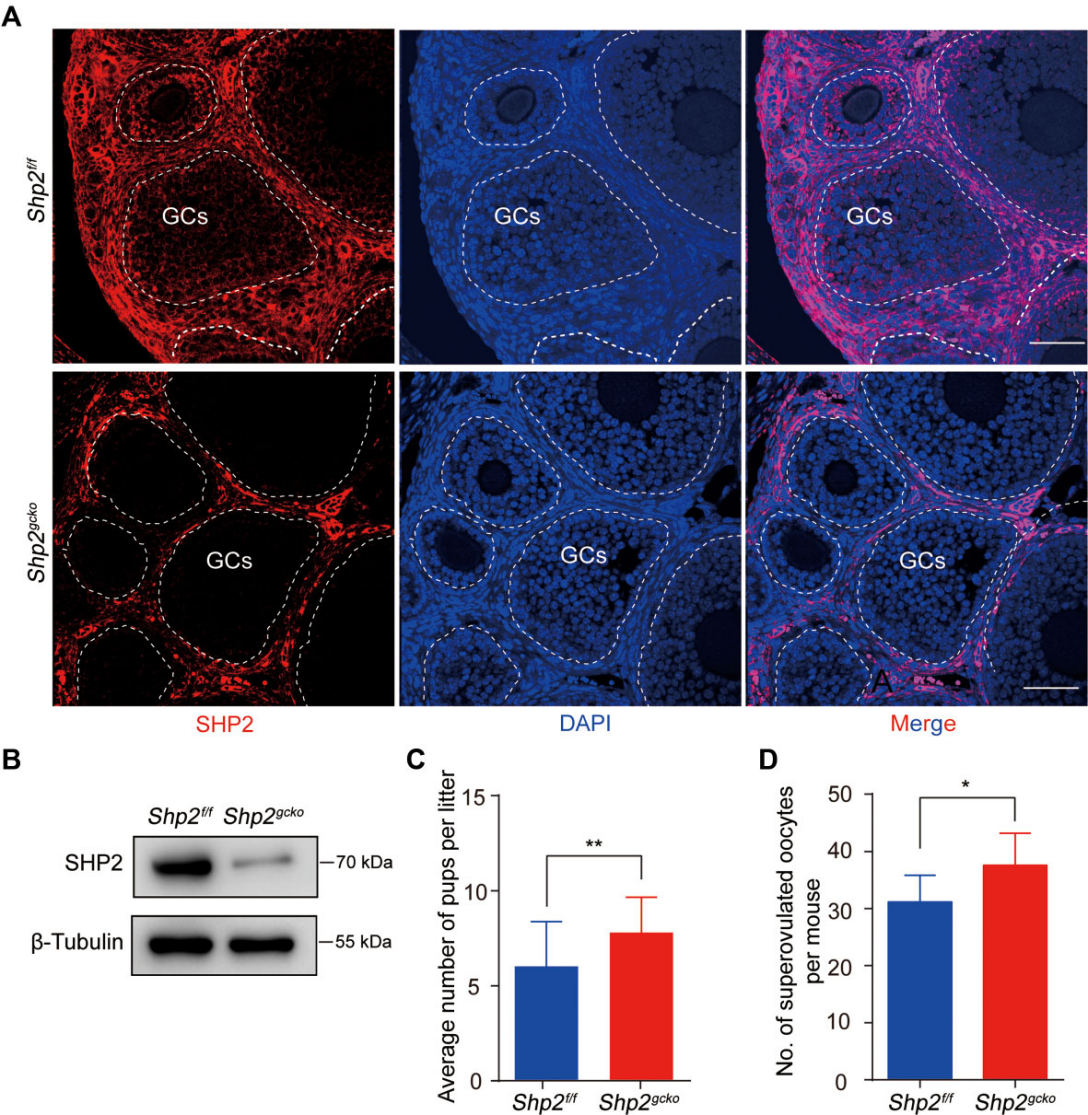
The effects of conditional knockout of *Shp2* in GCs on female fertility in mice. (**A**) SHP2 expression (red) was analyzed by immunofluorescence staining in the ovaries from the *Shp^f/f^* and *Shp2^gcko^* mice on postnatal Day 23. The GCs within follicles are indicated by white lines. The nuclei were counterstained with DAPI (blue). (**B**) SHP2 protein levels were measured by western blotting in mGCs isolated from follicles of the *Shp^f/f^* and *Shp2^gcko^* mice on postnatal Days 21–23 (*n* = 8). (**C**) The average pups per litter of either *Shp2^f/f^* or *Shp2^gcko^* females crossed with wild-type males in one-year continuous reproductive experiments (*n* = 8). (**D**) The number of superovulated oocytes from the *Shp2^f/f^* and *Shp2^gcko^* females at postnatal Days 21–23 after treatment with PMSG and hCG (*n* = 8). Scale bar, 50 µm. The data are presented as mean ± SEM from at least 8 mice in each group or three independent experiments. Statistical differences are indicated: *P < 0.05; **P < 0.01.

To identify the physiological role of SHP2 in ovarian GCs, we used a mouse model with GC-specific ablation of *Shp2* in developing follicles (*Shp2^gcko^*). As shown in Figure 1, SHP2 was observed in the cytoplasm of ovarian GCs in the *Shp2^f/f^* mice (Figure 1A, GCs in the white cycle in the upper panel) but was not detected in GCs of *Shp2^gcko^* ovaries (Figure 1A, GCs in the white cycle in the bottom panel). Then, we isolated and purified primary mouse ovarian GCs (mGCs) authenticated by immunofluorescence staining of FSH receptor (FSHR) (Supplementary Figure S1) and found that the SHP2 protein level in mGCs from the *Shp2^gcko^* group was extremely low compared with that of the controls (Figure 1B). These results demonstrated that *Shp2* was specifically and effectively ablated in the *Shp2^gcko^* mice.

To identify the physiological role of SHP2 in GCs in female reproduction, we performed a reproductive experiment by continuously crossing *Shp2^gcko^* female mice with wild-type male mice for one year. The average number of pups per litter of the *Shp2^gcko^* mice was significantly greater than that of the *Shp2^f/f^*female mice (Figure 1C, *P* < 0.01). Then, we performed a superovulation assay on immature mice, and the results showed that there were more oocytes from the *Shp2^gcko^* mice than that from the *Shp2^f/f^* group (Figure 1D). These results demonstrated that conditional ablation of *Shp2* in GCs enhanced female reproductive activity and oocyte production.

### Shp2 ablation increases follicular growth and inhibits follicular atresia in mice

**Figure 2:**
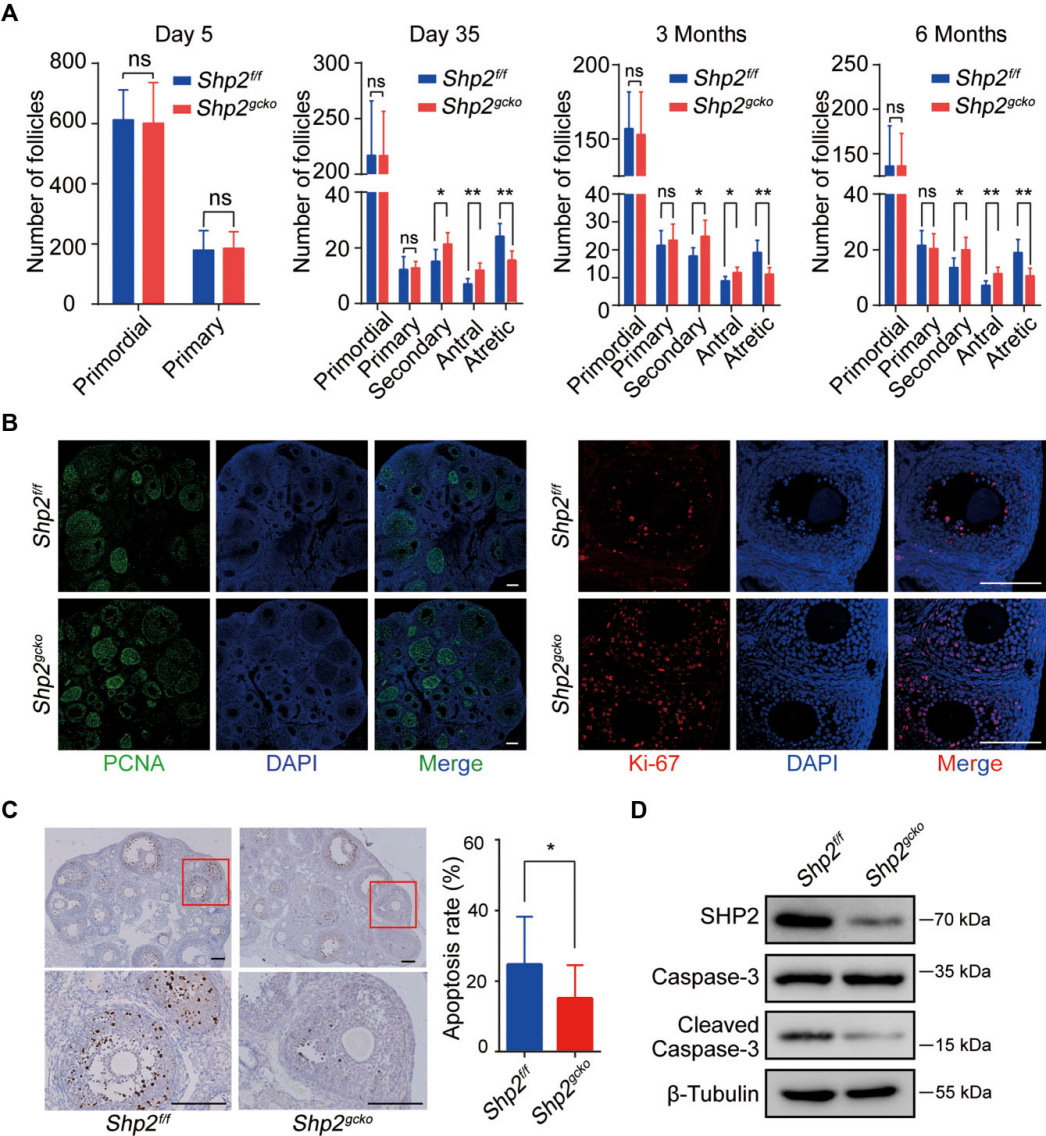
The effects of conditional ablation of *Shp2* on follicular development. (**A**) The respective numbers of primordial, primary, preantral, antral, and atretic follicles in ovaries of the *Shp2^f/f^* and *Shp2^gcko^* mice during estrus at the indicated time points. (**B**) The expression of the proliferation markers PCNA (left, in green) and Ki-67 (right, in red) was examined by immunofluorescence staining in the ovaries from the *Shp2^f/f^*and *Shp2^gcko^* mice at postnatal Day 35. The nuclei were counterstained with DAPI (blue). (**C**) Representative IHC staining images of cleaved Caspase-3 in the ovaries from the *Shp2^f/f^* and *Shp2^gcko^* mice at postnatal Day 35 (left) and the corresponding quantity statistics of apoptotic cells (right). (**D**) The expression of cleaved Caspase-3 and Caspase-3 in ovaries isolated from the *Shp2^f/f^* and *Shp2^gcko^* mice at postnatal Day 35. Scale bar, 100 µm. The data are presented as mean ± SEM from at least 8 mice in each group or three independent experiments. Statistical differences are indicated: *P < 0.05; **P < 0.01; ns, not significant.

The maturation of oocytes is achieved through follicular development, which determines the capacity of oocyte products and fertility in female mice. Therefore, we assessed the effects of *Shp2* deficiency in GCs on follicular development by counting the follicles at different stages (postnatal 5 days, 35 days, 3 months, and 6 months) in the ovaries from the *Shp2^f/f^* and *Shp2^gcko^* mice. The representative morphologies of the ovaries are presented in Supplementary Figure S2. At each stage, there was no difference in the number of primordial follicles and primary follicles between the *Shp2^f/f^* and *Shp2^gcko^* mice, which is consistent with *Shp2* beginning to be deleted in growing follicles with *Fshr-Cre*. However, there was a significantly higher number of secondary follicles and antral follicles in the *Shp2^gcko^* mice than in the *Shp2^f/f^* mice from 35 days to 6 months, demonstrating that *Shp2* deficiency promoted the growth of follicles. Interestingly, atretic follicles were significantly decreased in *Shp2^gcko^* ovaries from 35 days to 6 months, suggesting that *Shp2* ablation enhanced the survival of follicles (Figure 2A; Supplementary Figure S2). Together, these results illustrated that *Shp2* ablation improved follicular development by increasing the growth and survival of follicles.

To understand the cellular mechanism of SHP2 effect on follicular development, we evaluated cell proliferation in mouse ovarian tissue by two proliferation biomarkers, proliferating cell nuclear antigen (PCNA) and Ki-67, with immunofluorescence staining. The expression of PCNA was increased in *Shp2^gcko^* ovaries (Figure 2B; Supplementary Figure S3A). The number of Ki-67-positive GCs was significantly higher in *Shp2^gcko^* ovaries than in *Shp2^f/f^* ovaries (Figure 2B; Supplementary Figure S3B). Based on the expression of PCNA and Ki-67, which was focused on the area of GCs, these results suggested that *Shp2* deficiency promoted cell proliferation of GCs. Moreover, we evaluated the impact of SHP2 on cell apoptosis in follicles with immunohistochemistry (IHC) staining of cleaved Caspase-3, a cell apoptosis marker, and found that cleaved Caspase-3-positive cells were markedly decreased in ovaries from the *Shp2^gcko^* mice (Figure 2C). The level of cleaved Caspase-3 in *Shp2^gcko^* ovaries was also significantly lower than that in *Shp2^f/f^* ovaries (Figure 2D). These observations demonstrated that *Shp2* deletion inhibited the cell apoptosis of follicles, thereby improving follicular survival.

### SHP2 plays a negative role in the proliferation of GCs

**Figure 3:**
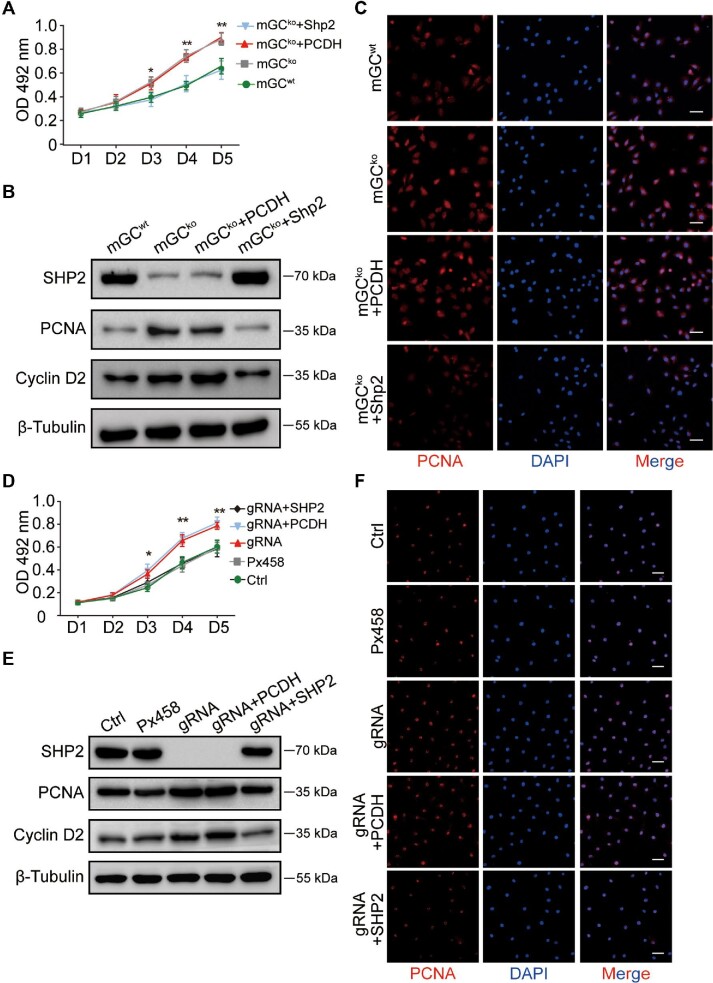
SHP2 ablation promotes cell proliferation of GCs *in vitro.* (**A**) The growth curves of mGCs with *Shp2* deletion were measured by MTT assays. (**B**) Western blotting analysis of SHP2, PCNA, and Cyclin D2 in mGCs with different treatments; β-Tubulin was used as the control. (**C**) PCNA (in red) expression in mGCs with *Shp2* deletion was examined by immunofluorescent staining. The nuclei were counterstained with DAPI (blue). (**D**) The growth curves of KGN cells with *SHP2* deletion were measured by MTT assays. (**E**) Representative protein levels of SHP2, PCNA, and Cyclin D2 in KGN cells with different treatments. β-Tubulin was used as the control. (**F**) PCNA expression (red) in KGN cells with *SHP2* deletion was examined by immunofluorescent staining. The nuclei were counterstained with DAPI (blue). PCDH, empty vector; *Shp2*, PCDH*-Shp2; SHP2*, PCDH*-SHP2*; Ctrl, control; *Px458*, empty plasmid; gRNA, *SHP2* knockout; OD, optical density. Scale bar, 50 µm. The data are presented as mean ± SEM from at least three independent experiments. Statistical differences are indicated: *P < 0.05; **P < 0.01.

To confirm the role of SHP2 in the proliferation of GCs, we assessed the proliferation of both mGCs and KGN cells with different protein levels of SHP2. mGCs were separated from *Shp2^f/f^* ovaries and *Shp2^gcko^* ovaries and further infected with lentivirus overexpressing *Shp2*. As shown in the top panel of Figure 3B, the expression level of SHP2 in the mGCs from the *Shp2^gcko^* mice (mGC^ko^) was much lower than that in the cells from the *Shp2^f/f^*mice (mGC^wt^) and recovered to a normal level after treatment with lentivirus overexpressing *Shp2* (mGC^ko^ + Shp2). As shown by the MTT assay, the primary mGCs exhibited a normal growth curve during 5 days of growth. However, the number of mGC^ko^ was markedly greater than that of mGC^wt^ on Days 3, 4, and 5. When intracellular SHP2 was rescued by transfection with lentivirus overexpressing *Shp2*, the growth rate was restricted to the basal level of the control cells (Figure 3A). With western blotting, we also observed that the removal of *Shp2* accelerated the expression of PCNA and Cyclin D2 in mGCs, and exogenous SHP2 curbed this biological effect (Figure 3B). Immunofluorescence staining also showed that *Shp2* knockout in mGCs powerfully increased the expression of PCNA, and exogenous SHP2 reduced the expression of PCNA to the basal level (Figure 3C).

Furthermore, we constructed an *SHP2* knockout KGN cell line (gRNA group) by CRISPR/Cas9 technology and observed that *SHP2*-null KGN cells presented a significantly higher growth velocity than the controls on Days 3, 4, and 5 (Figure 3D). Western blotting assays showed that exogenous *SHP2* eliminated *SHP2* deficiency-induced KGN cell proliferation and the expression of PCNA and Cyclin D2 (Figure 3E). Notably, *SHP2* knockout promoted the fluorescence intensity of PCNA (Figure 3F).

**Figure 4:**
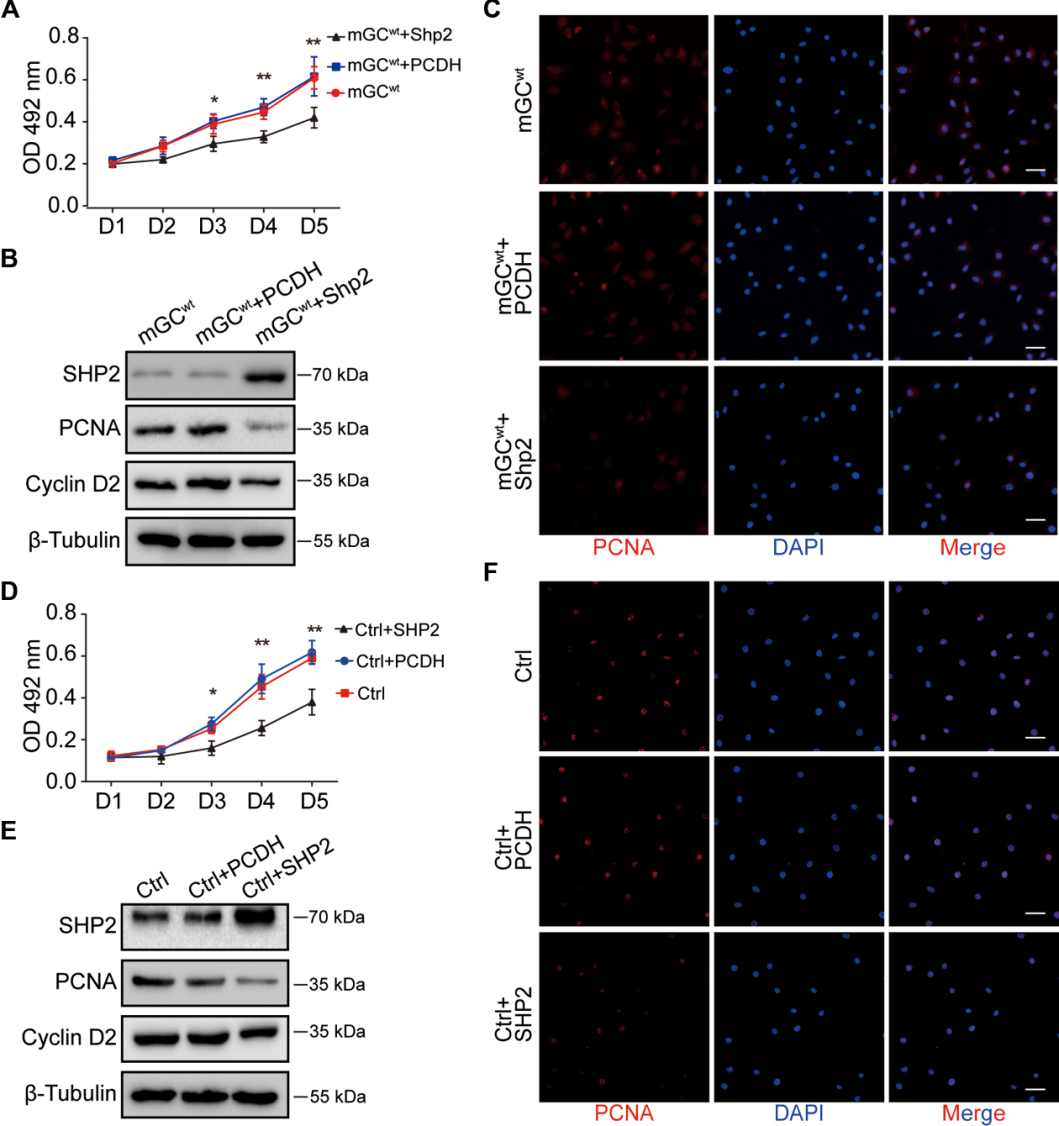
SHP2 overexpression inhibits the proliferation of GCs *in vitro.* (**A**) The growth curves of the mGCs overexpressing SHP2 were measured by MTT assays. (**B**) Representative protein levels of SHP2, PCNA, and Cyclin D2 in the mGCs overexpressing SHP2 were assessed by western blotting. β-Tubulin was used as the control. (**C**) PCNA expression (red) was measured by immunofluorescence staining in the mGCs overexpressing SHP2. The nuclei were counterstained with DAPI (blue). (**D**) The growth curves of the KGN cells overexpressing SHP2 were measured by MTT assays. (**E**) Representative protein levels of SHP2, PCNA, and cyclin D2 in the KGN cells overexpressing SHP2 were measured by western blotting. β-Tubulin was used as the control. (**F**) PCNA expression (red) was measured by immunofluorescence staining in the KGN cells overexpressing SHP2. The nuclei were counterstained with DAPI (blue). Scale bar, 50 µm. The data are presented as mean ± SEM from at least three independent experiments. Statistical differences are indicated: *P < 0.05; **P < 0.01.

In addition, we explored the effects of SHP2 overexpression on cell proliferation in mGCs and KGN cells. The results showed that the growth rate of mGCs and KGN cells overexpressing SHP2 was markedly attenuated (Figure 4A and D). The protein levels of PCNA and Cyclin D2 assayed by western blotting were decreased (Figure 4B and E), and the expression of PCNA assessed with immunofluorescence staining was significantly inhibited (Figure 4C and F), suggesting that SHP2 overexpression damaged the proliferation of mouse and human GCs. These observations suggest that SHP2 may antagonize cell proliferation in GCs.

### SHP2 accelerates apoptosis in GCs

**Figure 5:**
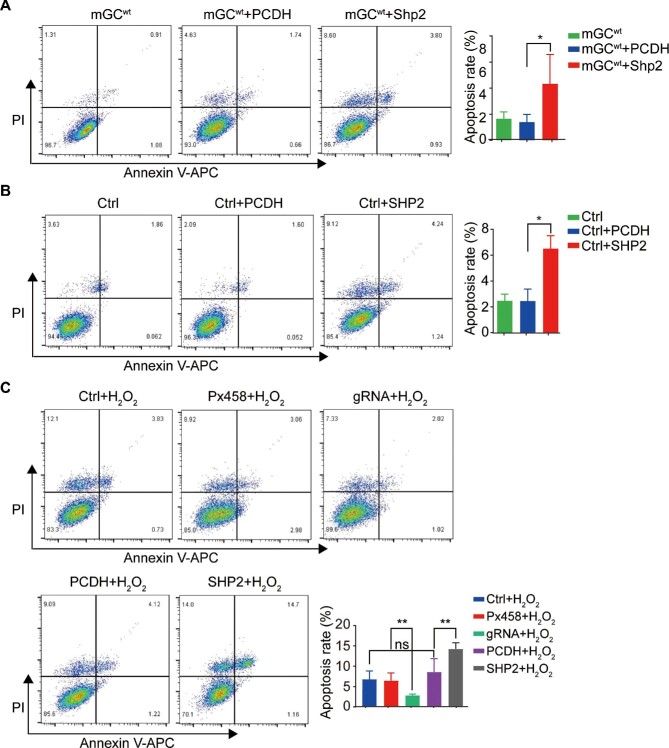
SHP2 accelerates cell apoptosis of GCs *in vitro.* (**A**) Representative images (left) and quantity statistics (right) of the apoptotic mGCs with SHP2 overexpression by flow cytometry. (**B**) Representative images (left) and quantity statistics (right) of the apoptotic KGN cells with SHP2 overexpression by flow cytometry. (**C**) Representative images (left) and quantity statistics (right) of the apoptotic KGN cells with SHP2 overexpression after H_2_O_2_ treatment by flow cytometry. The data are presented as mean ± SEM from at least three independent experiments. Statistical differences are indicated: *P < 0.05; **P < 0.01.

In addition to cell proliferation, we also analyzed the effects of *Shp2* deficiency on cell apoptosis. Because the apoptosis rate of GCs was low under basal conditions *in vitro, Shp2* ablation did not affect the apoptosis rate of mGCs (Supplementary Figure S4A and B). Apoptosis-related proteins (cleaved Caspase-3, Bax, Caspase-3, and Bcl-2) were relatively constant in the *Shp2*-deficient and normal mGCs (Supplementary Figure S4C). Similarly, in KGN cells, *SHP2* deletion did not affect cell apoptosis (Supplementary Figure S4D–F). However, the percentage of apoptotic mGCs overexpressing SHP2 was significantly increased (Figure 5A), which indicated that SHP2 overexpression enhanced the apoptosis of mGCs. SHP2 overexpression promoted the expression of Bax and cleaved Caspase-3 in mGCs (Supplementary Figure S5A). SHP2 overexpression in KGN cells also increased the percentage of apoptotic cells (Figure 5B) and the expression of Bax and cleaved Caspase-3 (Supplementary Figure S5B).

Numerous pieces of evidence indicate that oxidative stress plays a critical role in activating GC apoptosis. As a stimulant of oxidative stress, H_2_O_2_ is widely used in the study of GC apoptosis (Zhang et al., 2017). In this experiment, H_2_O_2_ effectively induced the apoptosis of normal KGN cells, and ablation of *SHP2* inhibited the H_2_O_2_-induced apoptosis of KGN cells, while overexpression of SHP2 promoted the apoptosis of KGN cells under H_2_O_2_ treatment (Figure 5C), which confirmed the pivotal role of SHP2 in cell apoptosis. These observations indicate that SHP2 positively regulates GC apoptosis and may play a balancing role in follicular development.

### SHP2 mediates cell proliferation by suppressing the PI3K/AKT signaling pathway

**Figure 6:**
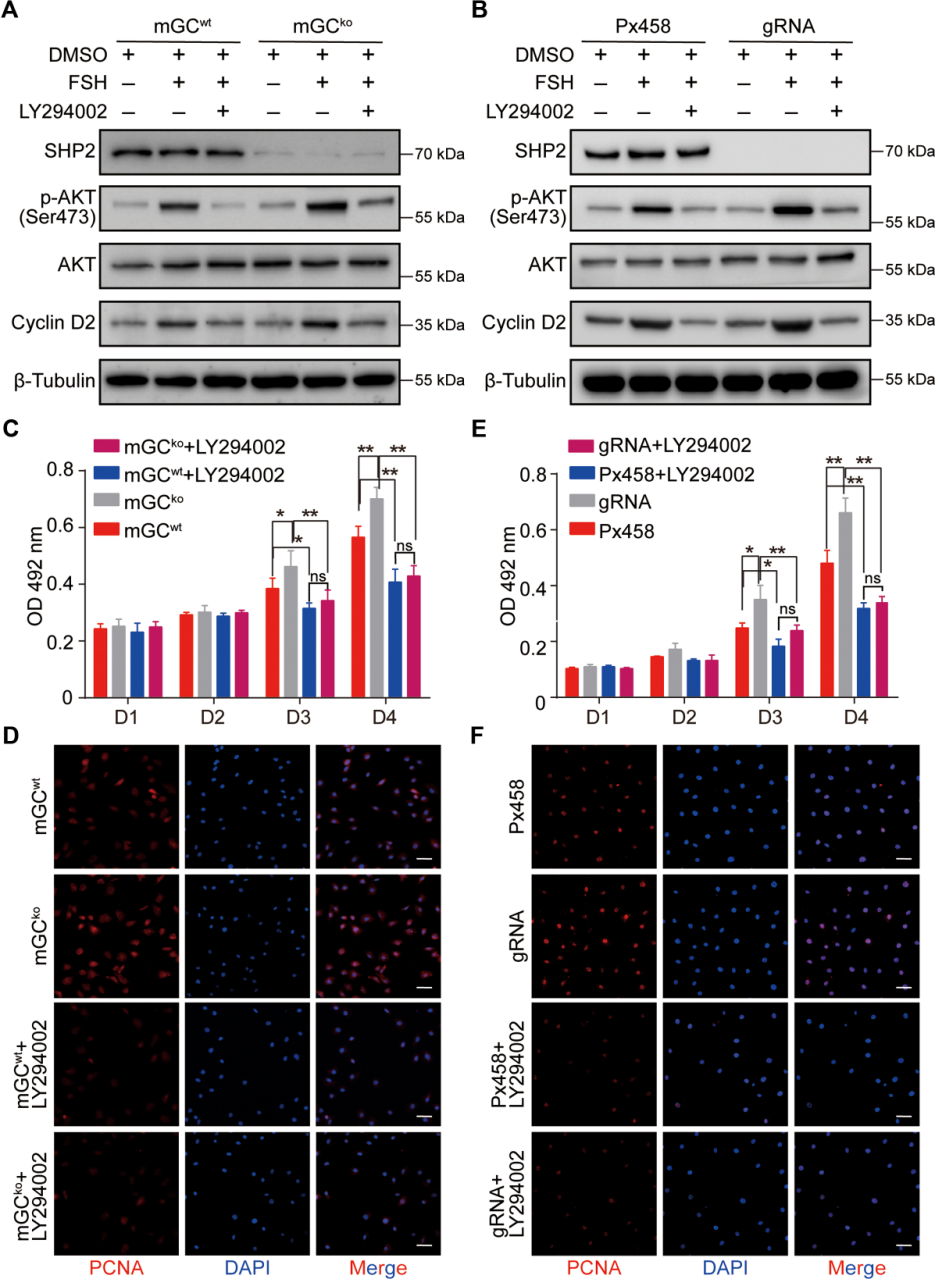
The role of SHP2 in GC proliferation is mediated by PI3K/AKT signaling. (**A**) Western blotting analysis of the expression of SHP2, p-AKT, and Cyclin D2 in the mGCs with SHP2 deficiency treated with LY294002. β-Tubulin was used as the control. (**B**) Western blotting analysis of the expression of SHP2, p-AKT, and Cyclin D2 in the KGN cells treated with LY294002. β-Tubulin was used as the control. (**C**) MTT assay for the growth of the mGCs treated with LY294002. (**D**) Immunofluorescence analysis of PCNA expression in the mGCs treated with LY294002. Red indicates PCNA and blue (DAPI) indicates the nucleus. (**E**) MTT assay for the growth of the *SHP2*-null KGN cells treated with LY294002. (**F**) Immunofluorescence analysis of PCNA expression in the *SHP2*-null KGN cells treated with LY294002. Red indicates PCNA and blue (DAPI) indicates the nucleus. Scale bar, 50 µm. The data are presented as mean ± SEM from at least three independent experiments. Statistical differences are indicated: *P < 0.05; **P < 0.01.

Next, we examined the effects of SHP2 on a key cytoplasmic signaling pathway, i.e. the PI3K/AKT pathway, in GCs with *Shp2* knockout. FSH potently induced the level of phospho-AKT (p-AKT) in normal GCs (mGC^wt^ group), but *Shp2* knockout (mGC^ko^ group) significantly increased the level of p-AKT. Pretreatment with LY294002, an AKT activation inhibitor, notably suppressed FSH-induced activation of AKT in mGCs either with or without SHP2 (Figure 6A). To test whether FSH could trigger *Cnot6* and *Cnot6l* expression in GCs derived from *Shp2^gcko^* mice, we synthesized specific primers for *Cnot6* and *Cnot6l* and performed quantitative reverse transcription–polymerase chain reaction (RT–PCR). The results showed that FSH could induce *Cnot6* and *Cnot6l* messenger RNA (mRNA) expression in mGCs, with a significantly higher level in the mGC^ko^ group than in the control (mGC^wt^) group (Supplementary Figure S6). In human KGN cells, deletion of *SHP2* also enhanced the FSH-induced phosphorylation of AKT, and LY294002 blocked the activation of AKT induced by both FSH and *Shp2* deficiency (Figure 6B). These observations suggest that SHP2 mediates FSH signaling by suppressing AKT activation.

The SHP2–AKT pathway was also observed to regulate the FSH-stimulated expression of Cyclin D2, a cell proliferation marker, in mGCs and KGN cells (Figure 6A and B), indicating that the SHP2–AKT pathway may be involved in FSH-induced proliferation of GCs. To confirm this hypothesis, we assessed the effect of LY294002 on FSH-induced cell proliferation of GCs with or without SHP2. Consistent with previous results, the growth rate of mGC^ko^ (Figure 6C, gray column) was markedly higher than that of the control mGC^wt^ (Figure 6C, red column) after incubation for 3 or 4 days (Figure 6C, *P* < 0.05). However, when mGCs were pretreated with LY294002, the number of *Shp2*-deficient cells was not notably increased compared with that of the control cells on Day 3 and Day 4 (Figure 6C, dark red column). *Shp2* ablation also facilitated the expression of PCNA, but LY294002 decreased the protein level of PCNA in both mGC^wt^ and mGC^ko^ (Figure 6D). Similarly, we observed that LY294002 strongly blocked the proliferation and PCNA expression of *SHP2*-null KGN cells (Figure 6E and F). The above results indicate that the PI3K/AKT pathway is involved in SHP2 regulation of GC proliferation.

### SHP2 is involved in H2O2-mediated apoptosis by regulating PI3K/AKT signaling

**Figure 7:**
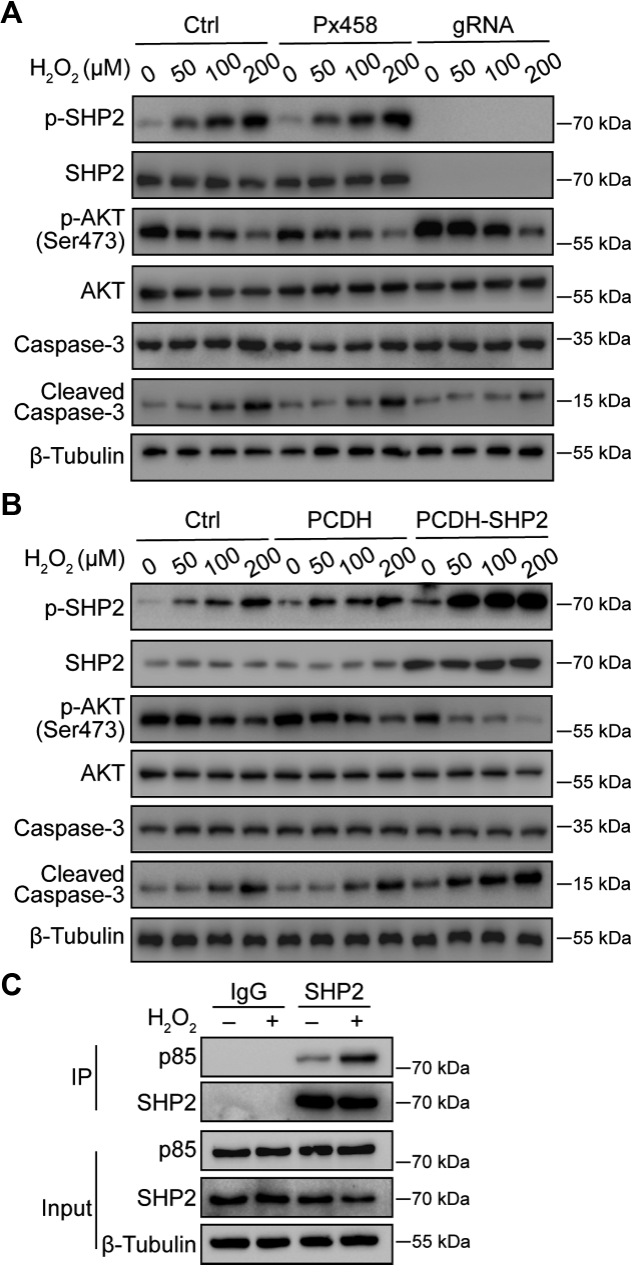
SHP2 is actively involved in H_2_O_2_-mediated GC apoptosis by regulating PI3K/AKT signaling. (**A**) Western blotting analysis of p-SHP2 and p-AKT expression levels in the *SHP2*-null KGN cells treated with H_2_O_2_. (**B**) Western blotting analysis of p-SHP2 and p-AKT expression levels in the *SHP2*-overexpressing KGN cells treated with H_2_O_2_. β-Tubulin was used as the control. (**C**) Interaction between SHP2 and p85 was determined by co-immunoprecipitation in the KGN cells with or without H_2_O_2_ treatment.

To clarify whether the PI3K/AKT pathway is involved in SHP2 regulation of GC apoptosis, we examined the activation of AKT in GCs with different protein levels of SHP2 under H_2_O_2_ treatment. In KGN cells, H_2_O_2_ was employed to induce apoptosis, as evidenced by the distinct observation of cleaved Caspase-3 in cells treated with 200 µM H_2_O_2_. *SHP2* knockout significantly inhibited whereas *SHP2* overexpression strongly promoted the cleavage of Caspase-3 induced by H_2_O_2_, which was consistent with the observation that SHP2 promoted KGN apoptosis. After treatment with H_2_O_2_, the p-AKT level decreased in KGN cells. However, the level of p-AKT was higher in the *SHP2* knockout cells but lower in the *SHP2*-overexpressing cells, compared with that in the normal control cells. Interestingly, H_2_O_2_ induced the phosphorylation of SHP2 (p-SHP2) in a dose-dependent manner (Figure 7A and B). More importantly, we found that SHP2 interacted with p85, a subunit of PI3K, and H_2_O_2_ enhanced the interaction between SHP2 and p85 in KGN cells (Figure 7C). These observations indicate that SHP2 modulates H_2_O_2_-stimulated PI3K/AKT signal transduction by binding to p85.

## Discussion

The present study demonstrated that specific deletion of SHP2 in ovarian GCs improved follicular development and oocyte production, which is associated with accelerating GC proliferation and inhibiting GC apoptosis.

In the mammalian ovary, follicular development is precisely regulated by endocrine and paracrine molecules via GCs to ensure the maturation of the oocyte (Li et al., 2010; Matsubara et al., 2019). Follicles begin to grow in response to gonadotropins during puberty (Grive and Freiman, 2015). *Fshr* mRNA transcripts were barely detectable, and none of the transcripts was detected full-length on postnatal Day 1 and Day 3. After Day 5, the full-length transcripts could be detected, and the full-length mRNA expression gradually increased on postnatal Day 7 and Day 10 (O'Shaughnessy et al., 1996). Thus, *Fshr-Cre* could not eliminate Shp2 in the GCs of primordial follicles. The full-length transcripts of *Fshr* only start to be expressed at a low level on postnatal Day 5 in the GCs of the primary follicle, and *Fshr-Cre* just begins to eliminate *Shp2* at this point, which may explain why there is no difference in primary follicles in the ovaries from the *Shp2^gcko^* and *Shp2^f/f^* mice. In brief, *Fshr-Cre* is an effective tool to delete *Shp2* in GCs of the secondary and antral follicles.

During follicular development, oocytes gradually accumulate a large number of materials, including mRNA and nutrients, to reach cytoplasmic maturity (Watson, 2007). In response to signals from the niche of follicles, oocytes themselves activate multiple signaling pathways to coordinate hormone and paracrine factors to control growth and maintain a synchronized growth rhythm (Watson, 2007; Li et al., 2010; Emori and Sugiura, 2014). As a result, the proper proliferation of GCs in follicles is important in orchestrating the growth of oocytes to prevent oocytes from either undernutrition or abnormal development (Watson, 2007) or triggering follicular atresia (Inoue et al., 2011; Regan et al., 2018). SHP2 ablation in GCs in growing follicles enhances follicular growth and inhibits follicular atresia, suggesting that SHP2 may be a negative signal protein to balance follicular development. Our study proved that SHP2 may act as a modulator to regulate the number of GCs within follicles that can be pivotal for oocyte maturation step by step.

The fate of follicles is closely associated with the proliferation and apoptosis of ovarian GCs (Matsuda et al., 2012; Regan et al., 2018). In the process of follicular growth, GCs dynamically undergo numerous changes, the most noticeable of which is the continuous proliferation of GCs (Braw-Tal, 2002; Kranc et al., 2017). Increasing evidence indicates that follicular atresia largely results from apoptosis of GCs, in which the imbalance between antioxidants and reactive oxygen is an important inducement (Yu et al., 2004; Inoue et al., 2011). Here, based on our *in vitro* studies, SHP2 ablation enhanced cell proliferation and inhibited H_2_O_2_-induced apoptosis, while overexpression of SHP2 suppressed cell proliferation and accelerated basal or H_2_O_2_-induced apoptosis. According to existing studies, SHP2 plays different regulatory roles in cell survival and proliferation in different kinds of cells. In many tissues, SHP2 promotes cell proliferation while suppressing cell apoptosis and thereby enhances tumor development (Zhang et al., 2015; Wang et al., 2021). In contrast, SHP2 inhibits cell survival behavior in liver cancer (Bard-Chapeau et al., 2011), colon cancer (Cai et al., 2014), metachondromatosis (Bowen et al., 2011), and esophageal squamous cell carcinoma (Qi et al., 2017) and facilitates cell death of glioma cells induced by co-inhibition of epidermal growth factor receptor (EGFR) and c-Met (Furcht et al., 2014). Our results indicated that SHP2 governs follicular development by regulating the proliferation and survival of GCs.

The PI3K/AKT pathway is a key cytoplasmic pathway in the activation of primordial follicles, follicular growth, and meiotic maturation of oocytes (Makker et al., 2014). FSH activates PI3K/AKT and its downstream cycle activator Cyclin D2 to promote GC proliferation (Park et al., 2005). In contrast, oxidative stress induced by peroxides, such as H_2_O_2_, significantly inhibits the PI3K/AKT pathway and induces apoptosis in GCs, which is closely associated with ovarian aging and female subfertility (Shen et al., 2017; Yao et al., 2020). During folliculogenesis, the PI3K/AKT pathway is accurately regulated by PTP, which can dephosphorylate PTK and thus govern ovarian function (Shimizu et al., 2009; Makker et al., 2014; Idrees et al., 2019). GC-specific disruption of *Pten* activates AKT to increase cell proliferation and reduce cell apoptosis of GCs, enhancing folliculogenesis and fertility in mice (Fan et al., 2008). Moreover, the *Kras^G12D^* mutant effectively disrupted *Pten* deletion-mediated follicular development, suggesting a conversation between the Ras/ERK and PI3K/AKT pathways in follicular development (Fan et al., 2009). CNOT6/6L-mediated mRNA deadenylation is essential for female reproductive endocrine regulation. FSH stimulates the transcription and translation of *Cnot6* and *Cnot6l* in ovarian GCs, which could function as key effectors of FSH in GCs and trigger the clearance of specific transcripts in GCs during the preantral-to-antral follicle transition (Dai et al., 2021). In this study, we found that FSH could also induce *Cnot6* and *Cnot6l* mRNA expression in mGCs, with a higher level in GCs from the *Shp2^gcko^* mice. These results suggest that SHP2 may also influence mRNA clearance in GCs through *Cnot6* and *Cnot6l* by regulating the PI3K/AKT pathway.

SHP2, as a nonreceptor phosphotyrosine phosphatase, dephosphorylates PTK (including RTKs, PI3K, and Gab1/2) to regulate the AKT signaling pathway in many tissues (Huang et al., 2014). SHP2 negatively regulates EGF-dependent PI3K activation and thus terminates the Gab1–PI3K positive feedback loop in fibroblasts (Zhang et al., 2002). Our study demonstrated that SHP2 negatively regulated FSH-dependent PI3K activation *in vivo* and *in vitro*, and blocking PI3K activation effectively reversed the SHP2 deficiency-induced proliferation. SHP2 may also regulate the AKT signaling pathway through some RTKs in GCs, e.g. IGF1 receptor showed a significantly increased transcript abundance and phosphorylation level in GCs treated with equine chorionic gonadotropin (Schuermann et al., 2018). In addition, through its phosphatase activity, SHP2 can positively or negatively regulate various cellular signaling pathways, such as the Ras/MAPK, Nrf2/NF-κB, and JAK/STAT signaling pathways. The Ras/MAPK signaling pathway is one of the most extensive intracellular signaling pathways and is involved in the regulation of cell proliferation, differentiation, survival, migration, and metabolism (Thatcher, 2010). The Nrf2/NF-κB signaling pathway regulates GC proliferation, enhances ER stress, induces cell cycle arrest, and mediates apoptosis after Pb-driven oxidative stress (Aglan et al., 2020). The JAK/STAT pathway may also be involved in regulating the proliferation and apoptosis of KGN cells (Huang et al., 2022). However, further investigation is required to explain whether SHP2 regulates the destiny of GCs and balances follicular development through these signaling pathways.

Moreover, many studies have shown that SHP2 is involved in cellular oxidative stress transduction and positively participates in oxidative stress-induced cellular damage (Gupta et al., 2012; Cai et al., 2018). In rat primary astrocytes, SHP2 phosphorylation was obviously induced with H_2_O_2_ treatment but blocked by reactive oxygen species (ROS) scavengers, which suggests that H_2_O_2_ mediates SHP2 phosphorylation by activating cellular ROS (Park et al., 2009). Importantly, the activity of SHP2 was also induced by oxidative stress, and SHP2 inhibition robustly attenuated brain injury induced by oxidative stress *in vivo* (Wang et al., 2012). In line with this, our study also demonstrated that SHP2 phosphorylation was activated by incremental H_2_O_2_, and Shp2 deletion alleviated whereas SHP2 overexpression promoted H_2_O_2_-mediated cell apoptosis by regulating PI3K/AKT signaling. Finally, we demonstrated that H_2_O_2_ promoted the binding between SHP2 and the regulatory subunit p85 of PI3K, suggesting that SHP2 may negatively regulate follicular development by suppressing PI3K/AKT.

In conclusion, our findings demonstrate that SHP2 may balance PI3K/AKT signaling to inhibit cell proliferation and promote apoptosis of ovarian GCs, thereby suppressing follicular development. The findings from this study identify SHP2 as a potential target for the treatment of ovarian dysfunction.

## Materials and methods

### Animal models and treatment

The generation of the conditional *Shp2* mutant allele (*Shp2^flox^*) in the C57BL/6 background mice was reported previously (Zhang et al., 2004). For the generation of animal models with selective deletion of *Shp2* in GCs, *Shp2^f/f^* mice were crossed with *Fshr-Cre* mice. The offspring inheriting both *Fshr-Cre* and two *Shp2* floxed deleted alleles were identified as knockout mice (*Shp2^gcko^*). *Shp2^f/f^* mice were used as controls. All mice were raised at Xiamen University Laboratory Animal Center according to the guidelines of the center. All animal experiments were performed according to the approved guidelines from the Animal Welfare Committee of Research Organization of Xiamen University. The genotypes of the mice were identified by PCR, and the primers used in this study are shown in Supplementary Table S1.

For evaluation of the reproductive ability of *Shp2^gcko^* mice, continuous reproductive experiments were performed for one year. Generally, 8-week-old *Shp2^gcko^* female mice (*n* = 8) and *Shp2^f/f^* control mice (*n* = 8) were crossed with one wild-type male mouse, respectively. The vaginal plug was detected to indicate mouse pregnancy. The number of pups was recorded to analyze fertility.

For the superovulation assay, 21 to 23-day-old female mice were injected intraperitoneally with 10 IU pregnant mare serum gonadotrophin (PMSG) per mouse for 48 h, followed by 10 IU human chorionic gonadotrophin (hCG) for an additional 18 h. The number of oocytes was counted after the ovulated cumulus–oocyte complex was digested by hyaluronidase.

### Hematoxylin–eosin, IHC, and immunofluorescence staining

Hematoxylin–eosin (HE), IHC, and immunofluorescence analyses were performed as previously described (Li et al., 2020). For IHC analysis, primary antibodies against cleaved Caspase-3 (Cell Signaling Technology, #9661), horseradish peroxidase (HRP)-conjugated secondary antibody (ZSbio, PV9001), and 3,3′-diaminobenzidine colorimetric reagent (ZSbio, ZLI-9018) were employed to detect the number of apoptotic cells. For immunofluorescence staining, primary antibodies against PCNA (1:200, Santa Cruz Biotechnology, sc-56) and Ki-67 (1:200, Cell Signaling Technology, #9129) and Alexa Fluor 555-labeled anti-rabbit (Beyotime, A0453), Alexa Fluor 555-labeled anti-mouse (Beyotime, A0460), and Alexa Fluor 488 anti-mouse IgG (ZSbio, ZF-0512) secondary antibodies were used to mark the proliferating cells. Slides were subsequently mounted with Vectorshield containing DAPI (H-1200, Vector Laboratories) and examined under a laser scanning confocal immunofluorescence microscope (LSM510 Exiter, Carl Zeiss).

### GC isolation, cell line construction, and treatment

For primary mGCs isolation, ovaries were removed from 21 to 23-day-old mice and punctured with 25-gauge needles. The cell suspension was collected and filtered with a 40-µm cell strainer to eliminate oocytes. mGCs were authenticated by immunofluorescence analysis using the FSHR antibody (Proteintech, 22665-1-AP).

The *SHP2* knockout KGN cells were engineered by CRISPR/Cas9 technology according to previous reports (Ran et al., 2017). SHP2-overexpressing adenovirus was added to *SHP2*-deficient cells and normal cells to restore or enhance the level of SHP2. All cells were cultured with DMEM/F12 medium (HyClone, SH30023.01) with 10% fetal bovine serum (Gibco, 10270), 100 IU/ml penicillin and 100 µg/ml streptomycin (HyClone, SV30010.01), and 10 mIU FSH (National Hormone and Peptide Program) in a 37°C humidified incubator with 5% CO_2_.

### MTT assay

KGN cells (1500 cells/well) or mGCs (4000 cells/well) were seeded in 96-well plates and allowed to continuously grow. MTT (Solarbio, IM0280) was added to the culture medium each day at the appointed time for incubation for 4 h followed by the addition of 100 µl of dimethyl sulfoxide to replace the medium. Absorbance was recorded at 492 nm with a Multiscan plate reader.

### Apoptosis assay

Cells were seeded in 6-well plates and treated with H_2_O_2_ (200 µM) or phosphate-buffered saline (PBS) as a negative control for incubation. Next, the cells were collected and washed twice with ice-cold PBS. After centrifugation at 500× *g* for 5 min, the cells were incubated in darkness for 10 min in 100 µl of ice-cold Annexin V binding buffer that contained 5 µl of Annexin V-APC, and then, 5 µl of propidium iodide (Thermo Fisher, 88-8007-72) was added for another 5 min. Eventually, cell apoptosis was analyzed with flow cytometry.

### Western blotting and co-immunoprecipitation

Proteins were extracted from mouse ovaries or cells by protein lysate containing 10 µg/ml protease inhibitor and 10 µg/ml phosphatase inhibitor. Then, western blotting assays were performed as previously described. Immunoblotting was performed with primary antibodies against AKT (Santa Cruz Biotechnology, sc-8312), p-AKT (Cell Signaling Technology, #4060), Caspase-3 (Santa Cruz Biotechnology, sc-56053), cleaved Caspase-3 (Cell Signaling Technology, #9661), Bax (Cell Signaling Technology, #2772), Bcl-2 (Cell Signaling Technology, #2876), p-SHP2 (Cell Signaling Technology, #5431), SHP2 (Santa Cruz Biotechnology, sc-280), p85 (Cell Signaling Technology, #4292), and Cyclin D2 (Abcam, ab3087). β-Tubulin (Proteintech, 66240-1-Ig) was used as a control. The grayscale of the western blotting bands was quantified by ImageJ (NIH).

For co-immunoprecipitation analysis, proteins were extracted as described above. After centrifugation at 12000× *g* for 15 min at 4°C, the supernatants were co-incubated with SHP2 antibody at 4°C overnight followed by incubation with protein A/G agarose beads for another 4 h according to the manufacturer's protocols.

### Assessment of follicular development

For evaluation of follicular development in the *Shp2^f/f^* and *Shp2^gcko^* mice, successive sections of the ovaries were made and subjected to HE staining. Every fifth section of the ovaries was used to count follicle populations. Only follicles with oocytes were counted to avoid double counting. Follicles were classified as primordial if they contained an oocyte surrounded by a single layer of flattened GCs or an incomplete layer of cuboidal GCs. Follicles were identified as primary if they contained an oocyte surrounded by a complete and single layer of cuboidal GCs. Second follicles were those containing an oocyte surrounded by 2–5 layers of GCs. Antral follicles were considered containing >5 layers of GCs. The follicles were considered to be atretic if their oocyte was degenerating or GCs had begun to disaggregate. The total number of follicles per ovary was determined by adding up all the counted sections of the whole ovary without a correction factor applied.

### Statistical analyses

All statistical analyses were performed with unpaired two-tailed Student's *t*-tests and two-way analysis of variance by using GraphPad Prism 6 software. All values are presented as mean ± standard error of the mean (SEM). Statistically significant differences were defined at *P ˂ 0.05 and **P ˂ 0.01.

## Supplementary Material

mjac048_Supplemental_File

## Notes

**Author contributions:** Z.L. and C.W. designed the research, analyzed the data, and revised the manuscript. X.W. performed the research, analyzed the data, and drafted the manuscript. L.Z. and Y.T. participated in the exploration of signaling pathways. H.W. helped in analyzing the data. Y.S. and G.F. provided genetic mouse model and analyzed the data. All authors read and approved the final manuscript.
