# Supplementary material for: Tyrosine phosphatase SHP2 in ovarian granulosa cells balances follicular development by inhibiting PI3K/AKT signaling
Source: J Mol Cell Biol. 2022 Aug 24;14(7):mjac048. doi: 10.1093/jmcb/mjac048 (PMC9764209; doi:10.1093/jmcb/mjac048)
Supplement: mjac048_Supplemental_File [file mjac048_supplemental_file.pdf]

Supplementary material

Tyrosine phosphatase SHP2 in ovarian granulosa cells balances follicular development by inhibiting PI3K/AKT signaling

Xiaoli Wei[1], Lanping Zheng[1], Yingpu Tian[1], Haibin Wang[2], Youqiang Su[3], Gensheng Feng[4], Chao Wang[5,*], and Zhongxian Lu[1,2,*]

[1] School of Pharmaceutical Sciences, State Key Laboratory of Cellular Stress Biology, Xiamen University, Xiamen 361005, China

[2] Fujian Provincial Key Laboratory of Reproductive Health Research, Medical College of Xiamen University, Xiamen 361102, China

[3] State Key Laboratory of Reproductive Medicine, Nanjing Medical University, Nanjing 211166, China

[4] Department of Pathology, Division of Biological Sciences, University of California at San Diego, La Jolla, CA 92093, USA

[5] State Key Laboratory of Agrobiotechnology, College of Biological Sciences, China Agricultural University, Beijing 100193, China

* Correspondence to: Zhongxian Lu, Tel/Fax: +86-592-2187227, E-mail: zhongxian@xmu.edu.cn; Chao Wang, E-mail: wangcam@cau.edu.cn

Running title: SHP2 in granulosa cells balances follicular development

Supplementary Table

Supplementary Table S1 Primer sequences for mouse genotyping and reverse transcription polymerase chain reaction.

Name of genes	Forward primer sequence	Reverse primer sequence	PrimerBank ID
Fshr-Cre	CTCTGGTGTAGCTGATGATC	TAATCGCCATCTTCCAGCAG	-
Cnot6	AACAGAACAACCACCTCCAAG	GTATAACTGCCGGGTCGCATA	29612526a1
Cnot6l	AAAATCTCACTGGGCAGAGTTAG	TGCGAGCAAGGTTATTGTCATT	121674806c1

Supplementary Figures

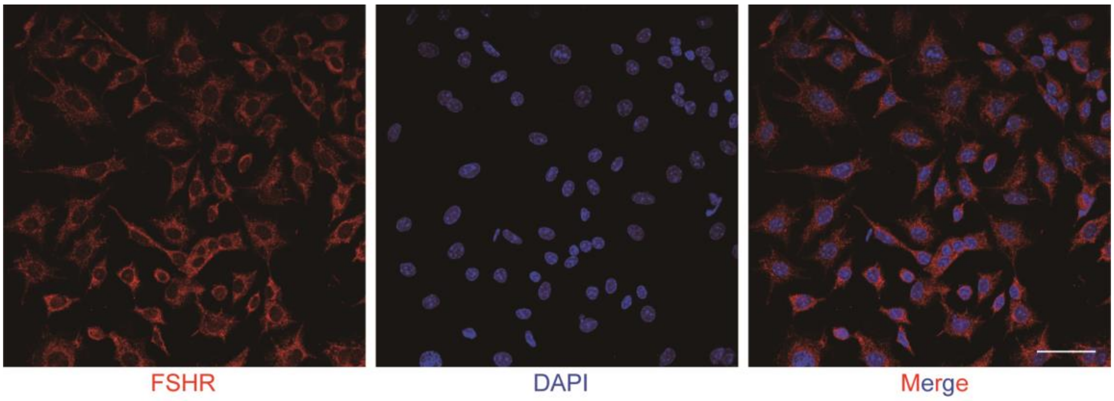

Supplementary Figure S1 Identification of mouse primary granulosa cells. Primary GCs were from postnatal day 23 *Shp*$^{f/f}$ mice and authenticated by FSHR antibody. Red fluorescence represented FSHR-positive cells. Scale: 100 μm.

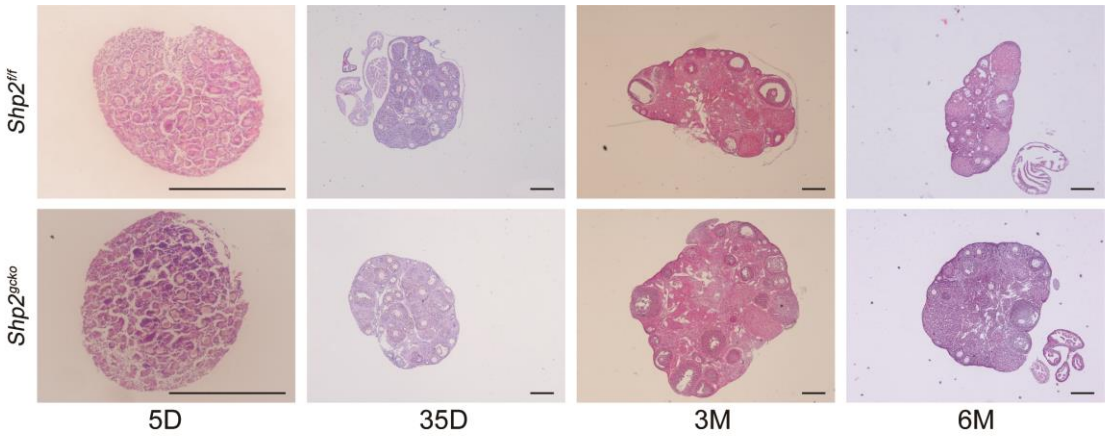

Supplementary Figure S2 Morphological detection of the ovaries in different stages. Ovarian morphology of the *Shp*$^{f/f}$ and *Shp2*^{gcko} mice from postnatal day 5, postnatal day 35, postnatal month 3, and postnatal month 6 were evaluated by HE staining. Scale bar: 100 μm.

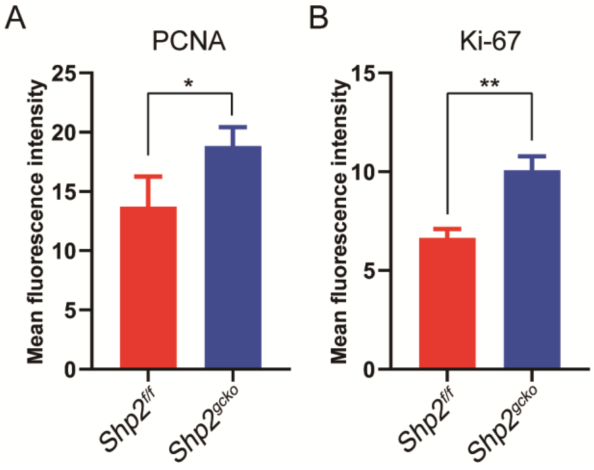

Supplementary Figure S3 The mean fluorescence intensity of PCNA and Ki-67 in the ovaries from the *Shp2*$^{f/f}$ and *Shp2*^{gcko} mice at postnatal Day 35. (**A**) The mean fluorescence intensity of PCNA in the ovaries from the *Shp2*$^{f/f}$ and *Shp2*^{gcko} mice at postnatal Day 35. (**B**) The mean fluorescence intensity of Ki-67 in the ovaries from the *Shp2*$^{f/f}$ and *Shp2*^{gcko} mice at postnatal Day 35.

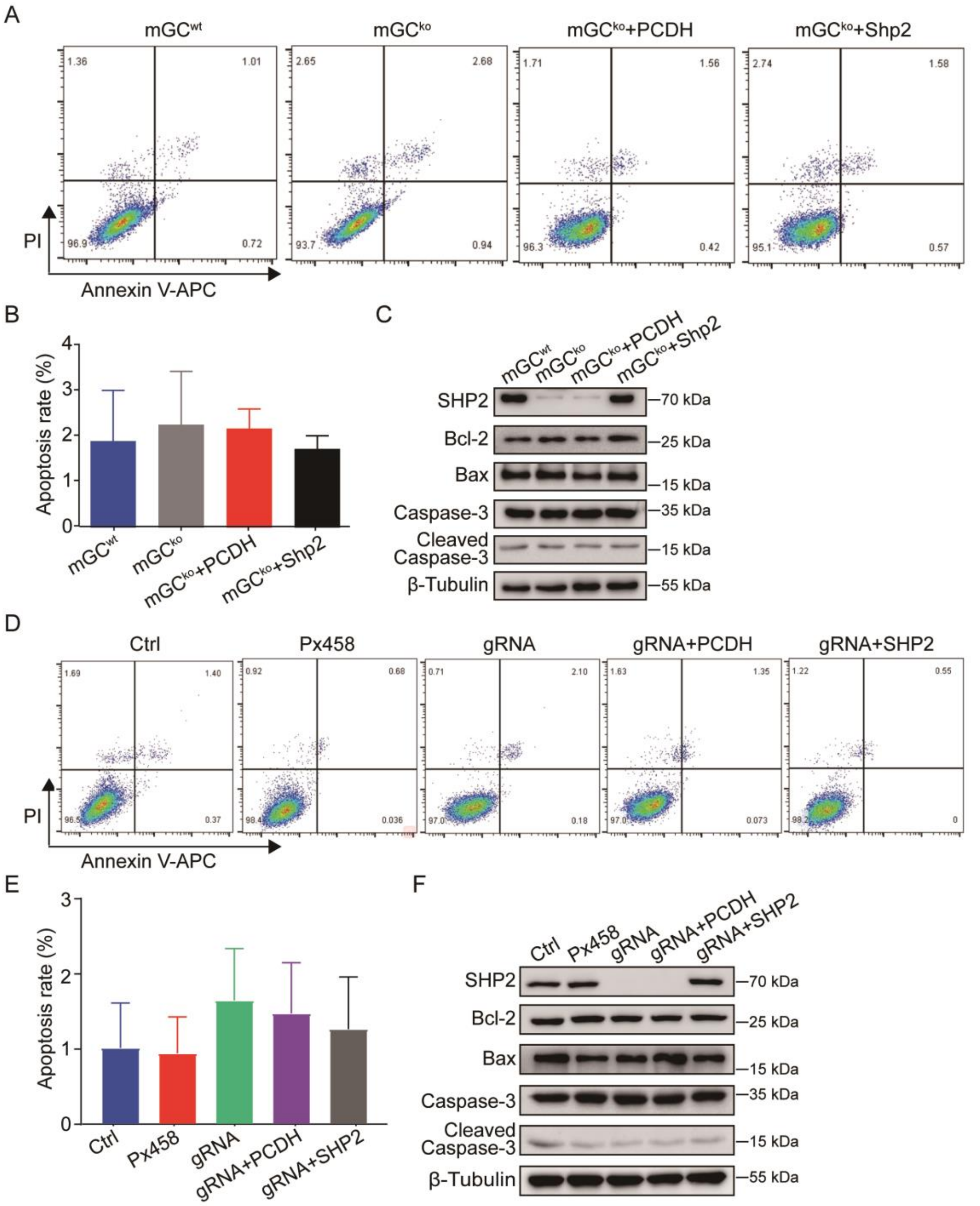

Supplementary Figure S4 Apoptosis detection in SHP2 deletion mGCs and KGN cells. (**A**) Apoptosis of *Shp2*-deficient mGCs was measured by flow cytometry. (**B**) The percentage of apoptotic cells in **A**. (**C**) The expression of apoptosis-related proteins was determined by Western blot. β-Tubulin was used as the control. (**D**) The apoptosis of *SHP2*-null KGN cells was measured by flow cytometry. (**E**) The percentage of apoptotic cells in **D**. (**F**) The expression of Bax, Bcl-2, and Cleaved Caspase-3 in four KGN cell lines.

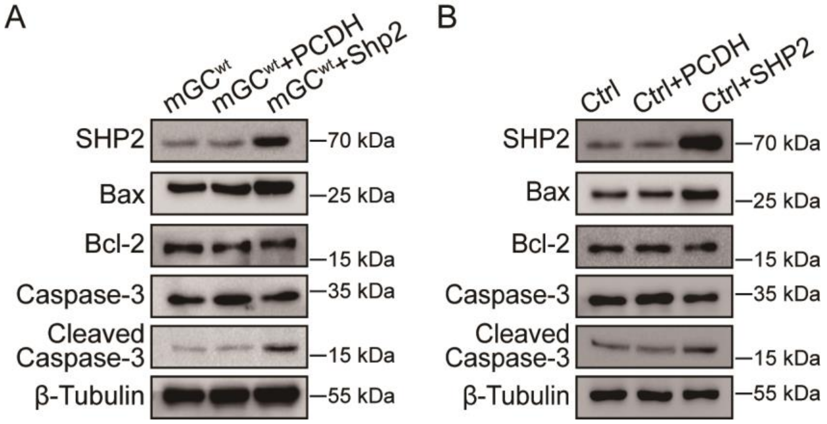

Supplementary Figure S5 SHP2 accelerates cell apoptosis of GCs *in vitro*. (**A**) Western blotting analysis of expression of apoptosis-related proteins in the mGCs overexpressing SHP2. β-Tubulin was used as the control. (**B**) Western blotting analysis of expression of apoptosis-related proteins in the KGN cells overexpressing SHP2. β-Tubulin was used as the control.

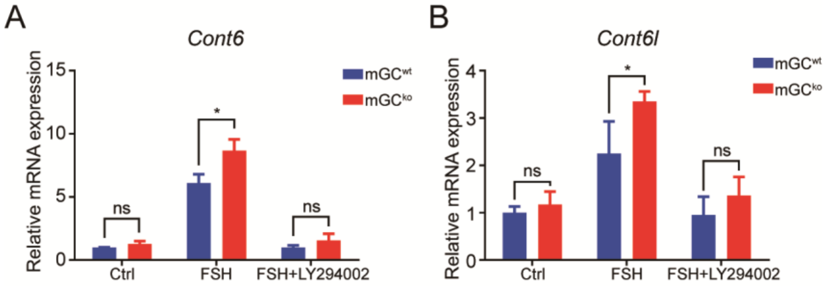

Supplementary Figure S6 *Cnot6* and *Cnot6l* mRNA expression in mGCs from the *Shp2*$^{f/f}$ and *Shp2*^{gcko} mice. (**A**) The mRNA expression of *Cnot6* in mGCs from the *Shp2*$^{f/f}$ and *Shp2*^{gcko} mice treated with FSH and LY294002. (**B**) The mRNA expression of *Cnot6l* in mGCs from the *Shp2*$^{f/f}$ and *Shp2*^{gcko} mice treated with FSH and LY294002.
